# Correlation study between serum uric acid level and cognitive impairment in middle-aged and elderly patients with hypertension: based on an explainable predictive model

**DOI:** 10.3389/fneur.2025.1708305

**Published:** 2025-11-19

**Authors:** Jiarui Wang, Hetong Liao, Ping Zheng, Qinfen Chen, Dongxu Lu, Yihong Huang, Di Wu, Jiajia Hu, Juan Du, Xiaoyun Zhang

**Affiliations:** 1 Department of Neurology, The Ninth Medical Center of Chinese PLA General Hospital, Beijing, China; 2 Department of Neurology, Tianjin Medical University General Hospital, Tianjin, China; 3 Department of Health Care, Liuzhou Workers Hospital, Liuzhou, Guangxi, China; 4 Department of Geriatrics, The First Affiliated Hospital of Wenzhou Medical University, Wenzhou, China; 5 The Northern Medical District, Chinese PLA General Hospital, Beijing, China; 6 Department of Internal Medicine, Beijing Water Conservancy Hospital, Beijing, China

**Keywords:** hypertension, serum uric acid, cognitive impairment, regression modeling, explainability, non-linear effects

## Abstract

**Background:**

Patients with primary hypertension are always comorbid with hyperuricemia. Serum uric acid exhibits a dual role in cognitive function. Evidence regarding the relationship between uric acid (SUA) and cognitive dysfunction in specific hypertensive patients remains inconsistent.

**Objective:**

To develop a predictive model to evaluate the association between serum uric acid level and mild cognitive impairment (MCI) in hypertensive populations.

**Methods:**

This cross-sectional study involved 420 middle-aged and elderly hypertensive patients. Cognitive function was evaluated using MMSE and MoCA. Univariate and multivariate logistic regression, restricted cubic splines (RCS), and SHAP analysis were employed.

**Results:**

In MCI group, diabetes prevalence, hyperuricemia prevalence, arteriosclerosis prevalence, education level, MMSE score, MoCA score, AD8 score and HbA1c were higher, while weight, BMI, SUA, TC, LDL, Alb, TT3, TSH, and FT3 were lower. After adjusting for confounding factors, it was found that the SUA level (OR = 0.754, 95%CI: 0.578–0.985, 0.038) could still be used as an independent protective factor for MCI. Subgroup analyses indicated effects varied significantly with diabetes history and regular exercise. Shap values showed that SUA is the fifth most related factor, with more significant ones including age, education level, albumin and thyroxin. A nonlinear association was found between SUA and MCI risk, with an inflection point at approximately 450 μmol/L.

**Conclusion:**

SUA has a certain correlation with MCI in the middle-aged and elderly hypertensive populations. Although SUA is considered as a neuroprotective agent, its neuroprotective function gradually diminishes and may even become detrimental when SUA higher than a threshold. These results suggest maintaining SUA within an optimal range may help mitigate MCI risk in hypertensive populations.

## 1 Introduction

The accelerating pace of aging has become an undeniable social trend, accompanied by a significant rise in the incidence of cognitive dysfunction disorders. Among these, dementia, particularly Alzheimer’s disease (AD), has emerged as a major challenge in the global public health domain that remains to be overcome. Epidemiological studies indicate that in developed countries, the prevalence of dementia among the elderly population aged 65 and above has climbed to 5–10% (1), with AD patients accounting for approximately half of these cases. Numerous factors related to dementia, including but not limited to genetics, gender differences, aging, educational level, lifestyle habits, and hypertension (2). Among these, hypertension is considered a significant controllable factor for cognitive dysfunction. As a common chronic disease, long-term uncontrolled hypertension can induce minor pathological changes in cerebrovascular structures and the blood–brain barrier (3, 4) through oxidative stress, exacerbating AD pathology and cognitive decline. Preventing cognitive dysfunction in hypertensive patients is of great significance. Identifying related risk factors for cognitive dysfunction and implementing effective prevention and treatment strategies will yield substantial clinical benefits.

Hyperuricemia is commonly observed in patients with primary hypertension, with relevant data indicating that approximately 38.7% of hypertensive patients also have increased serum uric acid (SUA) levels (5). Uric acid exhibits antioxidant properties, effectively inhibiting oxidative stress in neurons and providing protective effects for the brain (6). Some studies have found that subjects with gradually increasing SUA experience brain tissue atrophy, impaired microstructural integrity, and poorer cognitive function. However, decreased SUA levels are also proved associating with brain tissue atrophy (7). Nowadays, research on the relationship between uric acid and cognitive dysfunction remains limited, and a unified academic consensus has yet to be established. Most existing studies primarily focus on the general population, while discussions on the relationship between serum uric acid levels and cognitive impairment in specific groups, such as middle-aged and elderly hypertensive patients, remain relatively insufficient.

In this study, we constructed predictive model to assess the relationship between SUA and cognitive impairment using multimodal data, including ultrasound, MRI, and pathological indicators that are routinely collected in clinical settings.

## 2 Methods

### 2.1 Study cohort

This cross-sectional research analyzed clinical data from 434 middle-aged and elderly hypertension patients who visited the Physical Examination Center and Cognitive Clinic of the First Affiliated Hospital of Wenzhou Medical University between July 2023 and December 2024. This study was approved by the Ethics Committee of the First Affiliated Hospital of Wenzhou Medical University (KY2023-214). Inclusion criteria were as follows: (1) Age 45 years or older; (2) Meeting the diagnostic criteria for hypertension as defined in the *2024 Guidelines for the Prevention and Treatment of Hypertension* (8), systolic blood pressure ≥140 mmHg and/or diastolic blood pressure ≥90 mmHg, use of antihypertensive medication, or self-reported history of hypertension; (3) fully understood the purpose, procedures, and potential risks of this evaluation survey and voluntarily signed an informed consent form. Exclusion criteria included: (1) missing data over 20%; (2) presence of psychiatric abnormalities or neurological dysfunction that would prevent cooperation with scale assessments; (3) use of medications that may affect serum uric acid levels within the past month; (4) comorbid several central nervous system diseases or other systemic conditions affecting neurological function. Sample size estimation was performed based on the Events Per Variable (EPV) criterion for logistic regression (9).

### 2.2 Clinical data collection

General information of the study participants was collected through questionnaires, primarily including name, age, gender, education level, marital status, place of residence, exercise habits, dietary habits, past medical history, medication usage, family history of AD, smoking status, and alcohol consumption. Physical examination data were obtained during the health check-ups, including height, weight, waist circumference, hip circumference, and blood pressure. Body Mass Index (BMI) was calculated based on height and weight. Fasting venous blood samples were collected from all participants in the morning and analyzed by the Department of Laboratory Medicine at the First Affiliated Hospital of Wenzhou Medical University. Specific laboratory data refers to Table 1. All the patients underwent dual-energy X-ray absorptiometry (DXA), head magnetic resonance imaging(MRI), carotid artery ultrasound. DXA was used to assess bone metabolism status, categorizing patients into normal, osteopenia, or osteoporosis. Head MRI was used to rule out intracranial organic lesions while simultaneously assessing the presence of white matter changes. Carotid artery ultrasound was performed to evaluate arterial stiffness and exclude vascular malformations and stenosis.

**Table 1:** Baseline characteristics.

Characteristics	Non-MCI group (*N* = 139)	MCI group (*N* = 281)	Statistic	*P*-value
Gender (male)	66(47.48%)	133(47.33%)	0.001	0.977
History				
Hypertension	71(51.08%)	124(44.13%)	1.807	0.179
Diabetes	92(66.19%)	139(49.47%)	10.505	0.001*
Hyperlipemia	83(59.71%)	175(62.28%)	0.258	0.611
Family history of AD	20(14.39%)	27(9.61%)	2.138	0.144
Smoking	64(46.04%)	136(48.4%)	0.207	0.649
Drinking	78(56.12%)	170(60.5%)	0.739	0.390
Regular exercise	69(49.64%)	142(50.53%)	0.030	0.863
Hyperuricemia	103(74.1%)	233(82.92%)	4.519	0.034*
Arteriosclerosis	53(38.13%)	75(26.69%)	5.743	0.017*
SBI	42(30.22%)	97(34.52%)	0.778	0.378
Education			8.776	0.012*
Illiterate	42(30.22%)	104(37.01%)		
Primary school	59(42.45%)	134(47.69%)		
≥Middle school	38(27.34%)	43(15.3%)		
Preference for				
Sweet foods			3.987	0.136
Occasionally	17(12.23%)	19(6.76%)		
Rarely	58(41.73%)	115(40.93%)		
Daily/often	64(46.04%)	147(52.31%)		
Beverages			0.889	0.641
Occasionally	4(2.88%)	13(4.63%)		
Rarely	30(21.58%)	55(19.57%)		
Daily/often	105(75.54%)	213(75.8%)		
Coffee			3.753	0.153
Occasionally	5(3.6%)	7(2.49%)		
Rarely	31(22.3%)	43(15.3%)		
Daily/often	103(74.1%)	231(82.21%)		
Tea			0.371	0.831
Occasionally	11(7.91%)	27(9.61%)		
Rarely	31(22.3%)	59(21%)		
Daily/often	97(69.78%)	195(69.4%)		
Sleep duration			2.836	0.242
4–7 h	9(6.47%)	24(8.54%)		
> = 7 h	30(21.58%)	43(15.3%)		
≤4 h	100(71.94%)	214(76.16%)		
Exercise frequency			1.033	0.596
2–3 times per month	6(4.32%)	7(2.49%)		
1–7 times per week	14(10.07%)	29(10.32%)		
Never	119(85.61%)	245(87.19%)		
DXA			2.673	0.263
Normal	54(38.85%)	113(40.21%)		
Osteopenia	65(46.76%)	112(39.86%)		
Osteoporosis	20(14.39%)	56(19.93%)		
MMSE score	27(26, 28)	22(18, 26)	32388.000	0.000***
MOCA score	25(21, 26.5)	16(12, 20)	34034.500	0.000***
AD8 score	2(0, 3)	3(1, 4)	14108.500	0.000***
SCD9 score	6(4, 7)	6(4, 6.5)	−0.038	0.970
Age	55(50, 64)	62(54, 71)	13556.000	0.000***
Height	164(157, 169)	162(157, 169)	0.478	0.633
Weight	63.7(58.1, 72.4)	61(55, 69.3)	23089.000	0.002**
BMI	24.3(22.5, 26.25)	23.4(21.3, 25.3)	23408.500	0.001**
Waist circumference	87(81, 92)	86(79, 91)	1.319	0.188
Hip circumference	96(91.25, 100)	95(90, 99)	1.005	0.316
SHP	131(118, 144.5)	136(122, 150)	−1.715	0.087
DHP	78(70, 85.5)	79(72, 87)	−1.193	0.234
SUA	341(278, 394.5)	308(266, 375)	21947.000	0.039
VFA	79.3(56.5, 101.7)	75.2(53, 92)	1.165	0.245
HbA1c	5.8(5.6, 6.4)	6.3(5.8, 7.7)	12886.500	0.000***
TC	5.15(4.4, 5.9)	4.79(3.92, 5.7)	22578.500	0.009**
TG	1.36(1.06, 1.995)	1.4(1, 1.93)	−0.193	0.847
HDL	1.27(1.09, 1.47)	1.22(0.98, 1.44)	21604.000	0.076
LDL	3.18(2.645, 3.855)	2.91(2.26, 3.61)	22810.500	0.005**
Alb	43.6(41.85, 45.25)	42.1(38.7, 44.2)	25497.000	0.000***
Cr	70(58.5, 82)	65(55, 81)	0.033	0.973
eGFR	96.6(83.25, 104.55)	96.2(83, 105.3)	1.228	0.220
HCY	11(9, 13)	10.2(9, 13)	−0.512	0.609
Thyroxin	119.44(104.915, 133.405)	114.55(102.09, 130.75)	1.801	0.072
TT3	1.5(1.275, 1.76)	1.37(1.15, 1.57)	24067.000	0.000***
TSH	1.72(1.15, 2.26)	1.49(1.02, 2.2)	21426.000	0.105
FT	11.36(9.995, 12.245)	11.27(10.04, 12.53)	0.051	0.959
FT3	5.05(4.66, 5.495)	4.7(4.25, 5.27)	24856.000	0.000***
TGA	0.9(0.9, 0.9)	0.9(0.9, 0.9)	1.461	0.145
TPO	0.7(0.3, 1.45)	0.7(0.4, 1.4)	0.214	0.831
Non-HDL-C	3.8(3.1, 4.7)	3.5(2.7, 4.4)	0.527	0.598
LpoAI	1.37(1.255, 1.59)	1.35(1.19, 1.59)	1.382	0.168
LpoB	1.01(0.845, 1.285)	0.99(0.81, 1.27)	−0.267	0.790

*p < 0.05, **p < 0.01, ***p < 0.001.

The MMSE scale and the MoCA scale were used to assess the cognitive function of the participants. The participants had no prior exposure to these scales before the test. All evaluators received uniform training, and the testing environment was kept quiet. Both scales have a total score of 30 points. Cognitive impairment was defined as follows (10): an MMSE or MoCA score ≤17 for illiterate individuals, an MMSE or MoCA score ≤20 for those with primary school education, and an MMSE or MoCA score ≤24 for those with junior high school education or higher. Otherwise, cognitive function was considered normal. Meanwhile, AD8 scale and SCD9 scale were used.

### 2.3 Model development and interpretation

Python (Version 3.7) were used for model development, evaluation and visualizations. Due to the unbalanced outcomes, the research participants were stratified randomly divided into a training set and an internal validation set at a ratio of 7:3 to better the models. Performance metrics included the area under the receiver operating characteristic (ROC) curve (AUC).

To comprehensively evaluate the role of serum uric acid (SUA) in dementia and to gain a marginal understanding of its impact, we employed SHapley Additive exPlanations (SHAP) values. Mean absolute SHAP values enabled precise interpretation by quantifying the contribution of individual features to each patient’s risk. Variance contribution values quantifies each feature’s role in driving prediction variability across different samples. Convergence of these two independent metrics enhance clinical transparency and supporting personalized decision-making (10). Also, SHAP values were applied to rank the importance of input variables and interpret model effect. In contrast, SHAP values provide model interpretation with vectorial values, considering all possible combination of features.

### 2.4 Statistical analysis

Python 3.7 software was used for all the statistical analysis. The Kolmogorov–Smirnov method was used to test the normal distribution of the continuous variables. Then the mean ± sd of the measurement data was used for the normal distribution, and the student test was used for the comparison between the groups. Metrical data that did not conform to normal distribution was expressed as median (interquartile range) [M (P25, P75)]; and Mann–Whitney U test was used for comparison between groups. The Categorical variables were expressed by the number of cases and percentage (%), and the comparison between groups was carried out by Chi-square test. Statistically significant differences were defined as *p* < 0.05.

Then, collinearity analysis was performed on indicators that collectively influence dementia and showed statistically significant differences in univariate analysis, along with SUA. The Pearson correlation coefficient <0.7 was considered indicative of no collinearity among the independent variables. To evaluate the relationship between SUA and dementia, multivariate logistic regression analysis was conducted. Subgroup analysis was conducted to investigate the impact of the SUA on the risk of dementia across different populations. To explore the potential nonlinear relationship between SUA and MCI risk in the hypertension population, we used the restricted cubic spline (RCS) regression model to analyze the odds ratio (OR). The RCS model was implemented with 4 knots placed at the 5, 35, 65, and 95th percentiles of the SUA distribution (202, 295, 359.9, and 496.05 μmol/L, respectively).

## 3 Results

### 3.1 Characteristics of participants

A total of 553 middle-aged and elderly hypertensive patients were initially screened, of whom 420 met the inclusion criteria. Subjects were categorized by cognitive status into a mild cognitive impairment (MCI) group (*n* = 281) and a cognitively normal group (*n* = 139). Age ranged from 45 to 89 years, with a median of 60 (52, 69). In MCI group, diabetes prevalence, hyperuricemia prevalence, arteriosclerosis prevalence, education level, MMSE score, MoCA score, AD8 score and HbA1c were higher, while weight, BMI, SUA, TC, LDL, Alb, TT3, TSH, and FT3 were lower (*p* < 0.05). Table 1 summarizes the baseline characteristics.

### 3.2 Logistic regression analysis of the effect of SUA on MCI

**Figure 1:**
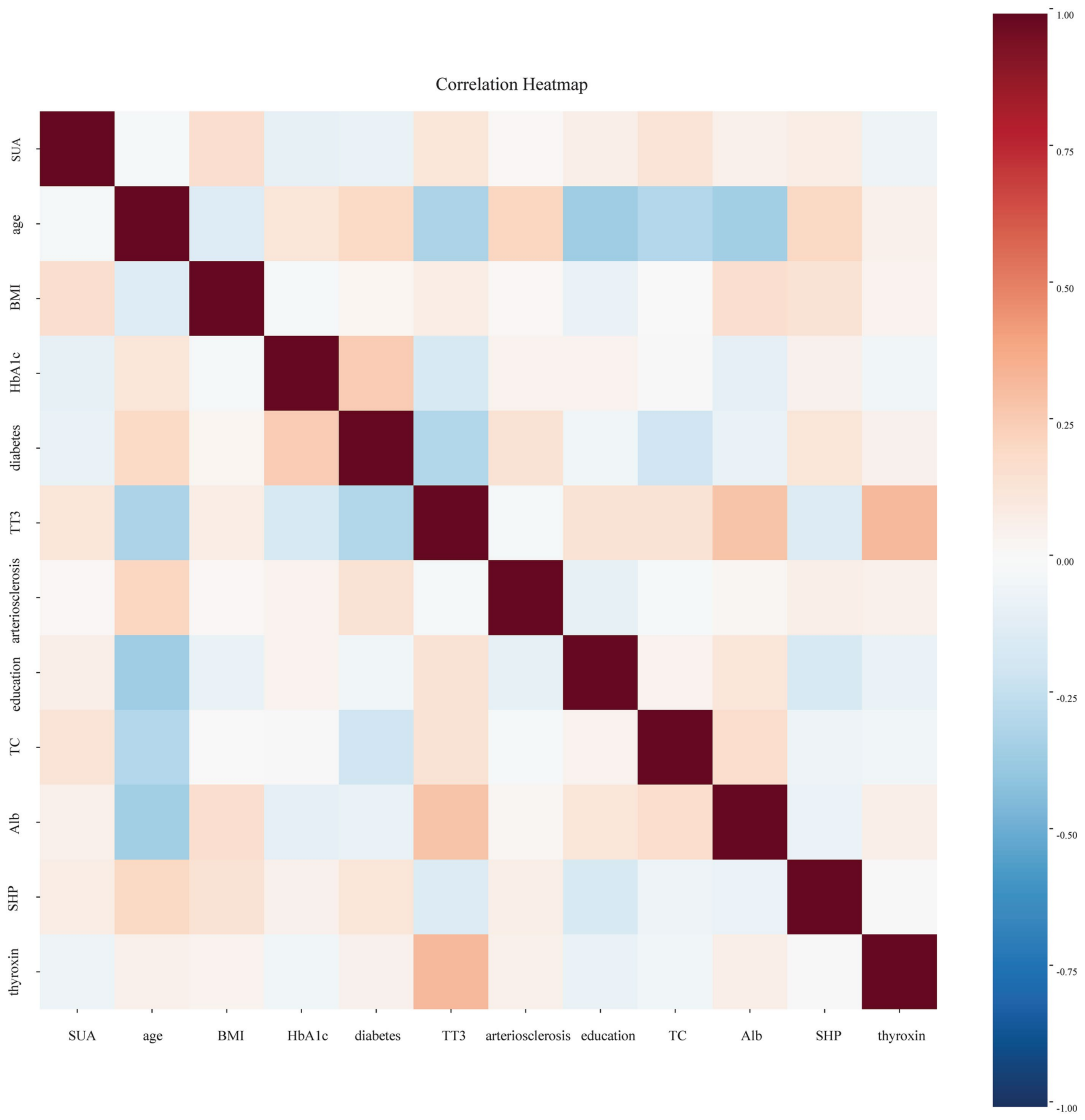
Correlation heatmap showing relationships between variables. The color gradient reflects the correlation coefficients, with darker shades indicating stronger correlations.

**Figure 2:**
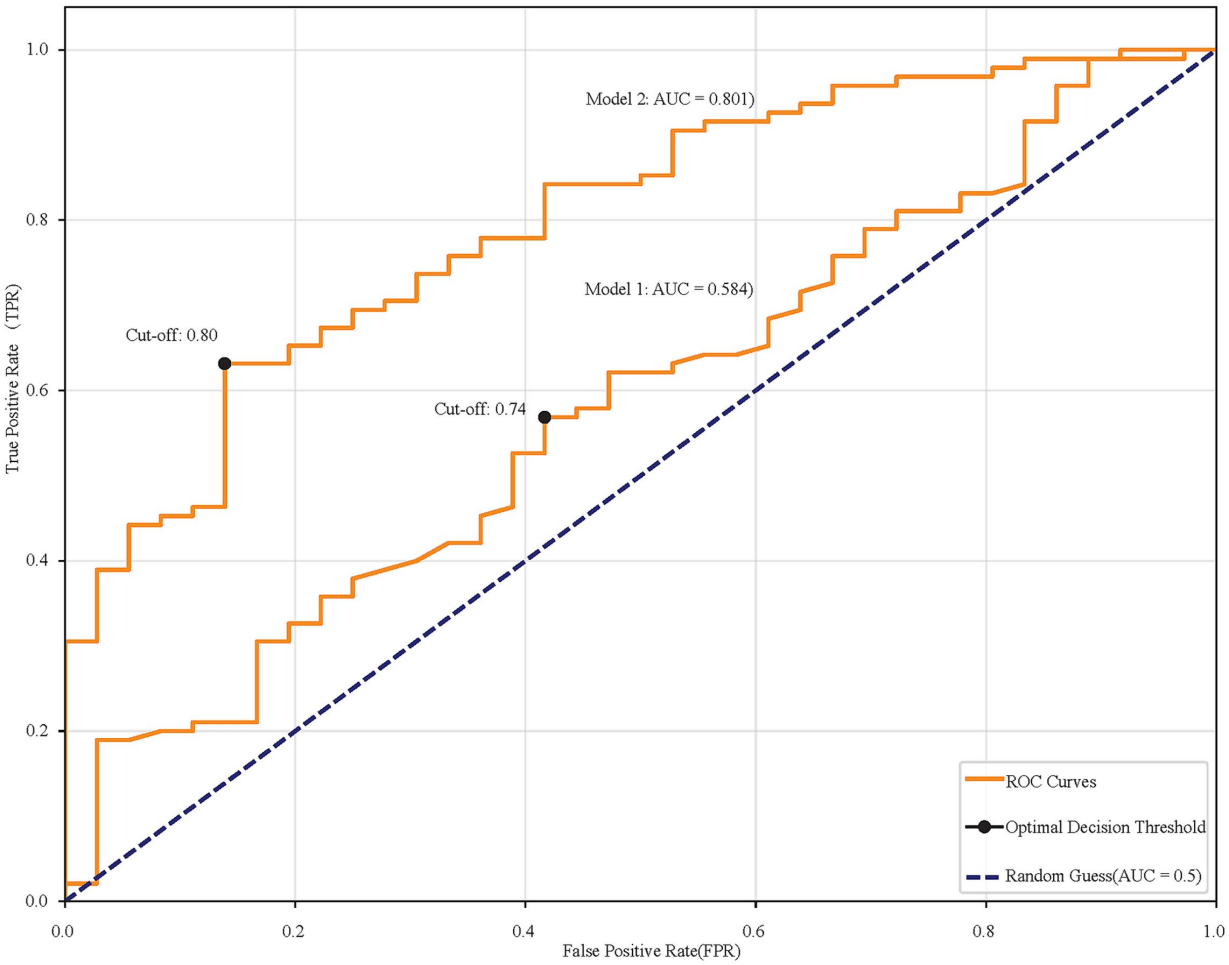
ROC curves comparing two models. Model 1 has an AUC of 0.584, and Model 2 has an AUC of 0.801. Cut-off points are marked at 0.74 and 0.80. The diagonal line represents random guessing with an AUC of 0.5.

Univariate analysis identified several related factors of MCI (*p* < 0.1), including history of diabetes, hyperuricemia, arteriosclerosis, education level, age, weight, BMI, SHP, HbA1c, TC, HDL, LDL, Alb, thyroxin, TT3, FT3. To avoid overfitting, correlated variables were removed using correlation analysis; a heatmap confirmed no strong correlations among retained variables (Figure 1). Twelve factors were incorporated into a multivariate logistic regression model with continuous variables standardized. The heatmap provided intuitive insights that there is no clear correlation between any two variables. When SUA was treated as a continuous variable, Model 1 (unadjusted) indicated that each standard deviation increase in SUA was associated with an 18.8% reduction in MCI risk (SUA: OR = 0.812, 95%CI: 0.670–0.985, *p* = 0.000). The original scale OR value converted from standardized results: SUA: OR = 0.99777 (per 1-unit increase). Model 2 (adjusted for confounding factors) showed a 24.6% risk reduction per SUA standard deviation increase (OR = 0.754, 95%CI: 0.578–0.985, 0.038). The original scale OR value converted from standardized results: SUA: OR = 0.99698 (per 1-unit increase). The results show in Table 2. ROC analysis was performed for each of the two models, and the resulting ROC curves are shown in Figure 2. The areas under the ROC curve were 0.801 and 0.584, respectively. A Hosmer-Lemeshow goodness-of-fit test was conducted for Model 2, which indicated good calibration (χ^2^ = 4.1636, *p* = 0.8421).

**Table 2:** Logistic regression model.

Characteristics	Model 1		Model 2	
	OR (95%CI)	*P*-value	OR (95%CI)	*P*-value
SUA	0.812 (0.670 ~ 0.985)	0.000	0.754(0.578 ~ 0.985)	0.038*
Quartile				
Q1	–	–	–	–
Q2	4.684(2.854 ~ 7.687)	0.000	2.150(1.069 ~ 4.323)	0.032*
Q3	1.946(1.309 ~ 2.893)	0.001	1.069(0.555 ~ 2.059)	0.841
Q4	1.892(1.270 ~ 2.818)	0.002	0.647(0.318 ~ 1.319)	0.231

*p < 0.05, **p < 0.01, ***p < 0.001.

When SUA was categorized by quartiles, the risk of MCI in the Q2, Q3, and Q4 group was significantly decreased compared to that in the lowest quartile (Q1) in Model 1 (unadjusted). In Model 2 (adjusted), compared to Q1, Q2 had significantly elevated MCI risk (OR = 2.150, 95%CI: 1.069 ~ 4.323, *p* = 0.032), while the higher quartile (Q3 and Q4) showed no significant difference (*p* > 0.05) (Table 2).

### 3.3 Subgroup analysis

Subgroup analyses and interaction tests were conducted based on the following stratifications: gender (male or female); history of diabetes (yes or no); history of hyperlipidemia (yes or no); family history of AD (yes or no); smoking history (yes or no); drinking history (yes or no); SBI (yes or no); regular exercise(yes or no); arteriosclerosis (yes or no); age (< 55 or ≥55); BMI (<23.9 or ≥23.9), SHP (<140 mmHg or ≥140 mmHg), DHP(<90 mmHg or ≥90 mmHg). Significant differences were observed between SUA and MCI across subgroups such as history of diabetes, family history of AD, drinking history, SBI, age and SHP (*p* < 0.05). Moreover, interaction effects were identified between SUA and history of diabetes and regular exercise (interaction *p* < 0.05). The results of the subgroup analysis are presented in Table 3.

**Table 3:** Interaction effect.

Subgroup variable	*P*-value	OR (95%CI)	Interaction P
Gender			0.909
Male	0.0714	0.545(0.282 ~ 1.054)	
Female	0.0930	0.513(0.236 ~ 1.117)	
History of diabetes			0.000***
Yes	0.086	2.969(0.858 ~ 10.267)	
No	0.000*	0.268(0.141 ~ 0.510)	
History of hyperlipemia			0.982
Yes	0.114	0.535(0.246 ~ 1.162)	
No	0.059	0.528(0.273 ~ 1.024)	
Family history of AD			0.977
Yes	0.363	0.520(0.127 ~ 2.128)	
No	0.022*	0.532(0.310 ~ 0.913)	
Smoking			0.707
Yes	0.088	0.576(0.305 ~ 1.086)	
No	0.078	0.471(0.204 ~ 1.090)	
Drinking			0.486
Yes	0.035*	0.477(0.240 ~ 0.950)	
No	0.345	0.689(0.318 ~ 1.494)	
SBI			0.993
Yes	0.041*	0.530(0.288 ~ 0.975)	
No	0.160	0.528(0.216 ~ 1.288)	
Regular exercise			0.033*
Yes	0.001*	0.300(0.146 ~ 0.615)	
No	0.833	0.924(0.440 ~ 1.939)	
Arteriosclerosis			0.226
Yes	0.051	0.587(0.343 ~ 1.003)	
No	0.054	0.207(0.042 ~ 1.026)	
Age			0.451
<55	0.028*	0.393(0.171 ~ 0.907)	
≥55	0.149	0.598(0.298 ~ 1.201)	
BMI			0.871
<23.9	0.269	0.621(0.266 ~ 1.445)	
≥23.9	0.087	0.568(0.297 ~ 1.085)	
SHP			0.600
<140 mmHg	0.021	0.469(0.247 ~ 0.891)	
≥140 mmHg	0.256	0.620(0.272 ~ 1.414)	
DHP			0.353
<90 mmHg	0.084	0.603(0.340 ~ 1.070)	
≥90 mmHg	0.051	0.336(0.113 ~ 1.003)	

*p < 0.05, **p < 0.01, ***p < 0.001.

### 3.4 Non-linear relationships between SUA and MCI risk

**Figure 3:**
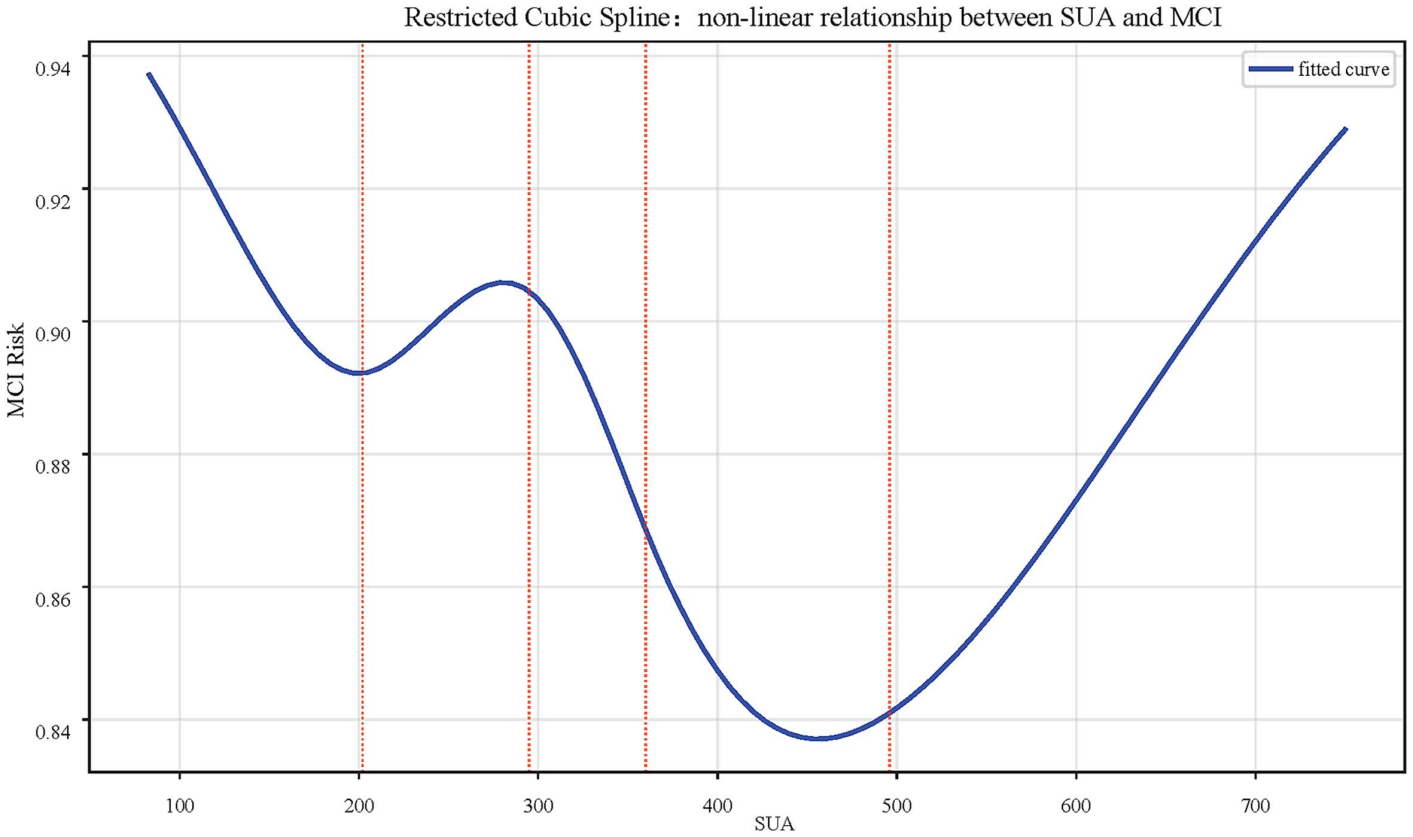
A restricted cubic spline showing a non-linear relationship between SUA and MCI risk. The curve dips initially, fluctuates with multiple peaks and troughs, and then rises sharply. Vertical dotted lines mark specific points on the SUA axis.

Multivariate logistic regression model using quartiles SUA indicated the relationship between SUA and MCI was influenced by multiple confounding factors and did not follow a simple linear pattern. To further investigate the potential nonlinear relationship, we employed a restricted cubic spline (RCS) model for fitting analysis and visualized the relationship. The results demonstrated that the association between SUA and MCI risk exhibited significant nonlinear characteristics with a critical inflection point observed at approximately 450 μmol/L. When SUA levels were below 450 μmol/L, higher SUA levels were associated with lower MCI risk. However, when SUA levels exceeded this threshold, this protective relationship was reduced and reversed, with elevated SUA levels becoming significantly associated with increased MCI risk (Figure 3).

### 3.5 Model interpretation with SHAP

**Figure 4:**
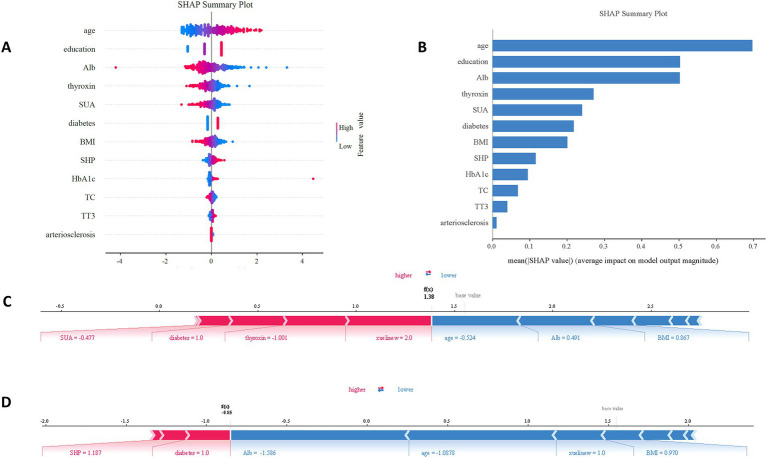
SHAP interpret the model. **(A)** Attributes of characteristics in SHAP. Each line represents a feature and the abscissa is the SHAP value. Red dots represent higher eigenvalues and blue dots represent lower eigenvalues. **(B)** Feature importance ranking as indicated by SHAP, showing the importance of each covariate in the development of the final predictive model. **(C)** Individual efforts by patients with MCI and **(D)** without MCI. The SHAp value represents the predicted characteristics of an individual patient and the contribution of each characteristic to the MCI. The number in bold is the probability forecast value [f(x)], while the base value is the predicted value without providing input to the model. F(x) is the logarithmic ratio of each observation. Red features indicate an increased risk of MCI and blue features indicate a reduced risk of MCI. The length of the arrows helps visualize the extent to which the prediction is affected. The longer the arrow, the greater the effect.

SHAP were used to quantify the contribution of each feature to the risk model. Global interpretability was illustrated using SHAP summary bar plots (Figure 4A), which ranked features by their average importance: age, education, Alb, thyroxin, SUA. SHAP summary scatter plots (Figure 4B) further visualized how the magnitude and direction of feature values related to outcomes. Mean Absolute SHAP Values and Variance Contribution showed in Table 4. And a strong consistency in the ranking of features between these two metrics were observed, indicating that the model could identify clinically relevant features stably.

**Table 4:** SHAP analysis.

Feature	Variance contribution (%)	Mean absolute SHAP value (%)
Age	36.34	23.65
Education	24.04	17.42
Alb	16.61	16.83
Thyroxin	6.64	9.39
SUA	5.01	8.00
HbA1c	3.60	2.33
BMI	3.51	6.84
Diabetes	2.67	7.53
SHP	1.09	3.91
TC	0.35	2.17
TT3	0.13	1.39
Arteriosclerosis	0.03	0.52

Age exhibits the largest change in SHAP values, indicating it is a significant correlation with occurrence of MCI. Its high feature values (red points) correspond to positive SHAP values, suggesting a positive correlation with the outcome. The feature SUA shows negative SHAP values at high feature values (red points) but positive SHAP values at lower values (blue points), indicating an overall negative effect of SUA on the MCI model. Additionally, two typical examples are provided to illustrate the interpretability of the model. One example pertains to patients who developed MCI with a higher SHAP score (0.800) (Figure 4C), while the other example involves patients who did not develop MCI, exhibiting a lower SHAP score (0.299) (Figure 4D).

## 4 Discussion

In this study, a cross-sectional cohort analysis was conducted involving 420 middle-aged and elderly hypertensive patients, among whom 281 (66.9%) were assessed as having mild cognitive impairment (MCI). Preliminary univariate analysis and logistic regression model indicated a significant association between SUA and MCI, and SUA levels possess certain predictive value for the risk of MCI occurrence. SHAP value analysis revealed it to be the fifth most significant related factor. When SUA was treated as a continuous variable, particularly after adjusting for all confounding factors, an increase in SUA was negatively correlated with the risk of developing MCI (OR = 0.754, 95% CI: 0.578–0.985, *p* = 0.038). When constructing a multivariate logistic regression model using quartiles, we found that the relationship between SUA and MCI was significantly influenced by multiple confounding factors such as age, BMI, and diabetes status; and may be nonlinear relationship. Then, SUA was found to have a dual effect on MCI risk by the RCS analysis. The closer SUA is to this threshold, the stronger its protective effect on brain cognitive function. However, when SUA exceeds this threshold, its neuroprotective function gradually diminishes and may even become detrimental. This study identified SUA as an important independent factor for MCI occurrence in middle-aged and elderly hypertensive populations.

Hypertensive patients often comorbid with hyperuricemia. The promoting effect of hypertension on cognitive dysfunction may occur through oxidative stress, which induces biological changes in endothelial cells, disrupts the blood–brain barrier, and consequently reduces the clearance of soluble beta-amyloid oligomers from the central nervous system (11). This process promotes neuroinflammation and neurodegenerative changes, exacerbates Alzheimer’s disease (AD) pathology, and accelerates cognitive decline. In this study, only the second quartile (Q2) of SUA shows a significant protective association against MCI compared to the lowest quartile (Q1), while the Q3 and Q4 do not. Then, we hypothesize that this may indicate a “U-shaped” or “J-shaped” relationship between UA and MCI risk. At very low levels (Q1), the lack of UA’s antioxidant benefits might increase risk. At moderate levels (Q2), the neuroprotective antioxidant effects may be optimal. However, at higher levels (Q3, Q4), the potential pro-oxidant, pro-inflammatory, or vascular risks of hyperuricemia might counteract and eventually negate its protective benefits.

Previous findings on the relationship between serum uric acid (SUA) levels and dementia, especially AD are often inconsistent. However, understanding the effect of SUA on MCI is critically important for preventing cognitive dysfunction. These divergent results imply a potential non-linear relationship between SUA and MCI (12), necessitating the application of more explainable and visually interpretable analytical methods to address this question. Nonetheless, standard logistic regression typically only reveals the global and average effects of variables. To overcome this limitation and comprehensively elucidate the complex relationship between SUA and MCI, this study innovatively integrates two advanced techniques — SHapley Additive exPlanations (SHAP) values and Restricted Cubic Splines (RCS), which are using widely in dementia risk researches (13, 14). This combined approach aims to provide the most thorough and in-depth interpretation of SUA’s role from both global importance and local relationship morphology.

Then, uric acid is considered a double-edged sword, with studies suggesting a U-shaped relationship between SUA levels and neurological disorders (15). On one hand, SUA acts as a neuroprotective agent. It suppresses the neuroinflammatory cascade by modulating immune cell activity and inhibiting the release of inflammatory factors, thereby reducing neuronal damage. As an endogenous antioxidant, SUA scavenges oxygen free radicals and other reactive radicals in plasma, blocking oxidative chain reactions that confer neuroprotective properties (16). On the other hand, when SUA concentrations exceed physiological ranges, abnormally elevated uric acid transforms into a pro-oxidant within cells, exacerbating oxidative stress-induced cellular damage. Simultaneously, inflammation factors induced by high SUA levels promote increased deposition of Amyloid *β*-protein (Aβ) in the hippocampus through multiple molecular pathways (17), accelerating central neurodegenerative changes that ultimately adversely affect cognitive function. Furthermore, elevated SUA often accompanies metabolic syndrome, subsequently leading to issues such as vascular endothelial dysfunction (18). Recent research has shown that in populations with metabolic syndrome and SUA levels >400 μmol/L, the risk of all-cause dementia also increases (19). A Mendelian randomization study demonstrated that per standard deviation increase in SUA levels (1.33 mg/dL), the risk of Alzheimer’s disease increases by 0.09 (OR: 1.09, 95% CI: 1.01–1.18) (20). However, considering the antioxidant properties of uric acid, excessively low serum uric acid levels may reduce the body’s resistance to oxidative stress and damage. When SUA levels are too low, the UA antioxidant and iron scavenger features diminish (21), potentially exacerbating cognitive impairment. A prospective cohort study (22) enrolled 17,707 participants across 28 provinces in China and investigated the longitudinal association between baseline serum uric acid levels and cognitive function over the 7-year follow-up. Their results showed that higher baseline SUA levels were negatively associated with cognitive decline (indicating a protective effect), but this protective effect disappeared when SUA levels became excessively high. They also found that the protective effect of SUA disappeared in females, when they were complicated by cardiometabolic disease. Although we find that gender had no interaction effect in the relationship between SUA and MCI, females have been found have more superior plasmatic antioxidant defenses than males (23). Another study in Asian population also showed that female MCI patients may have higher serum uric acid, alleviating longitudinal metabolic changes and cognitive decline (24). Previous research indicates that race does not significantly affect CSF metabolite levels within the purine pathways (25). This suggests that the biological relationship identified in our study might be consistent across different racial backgrounds. However, as our study cohort was predominantly of Han Chinese ethnicity, the generalizability of our specific predictive model to other populations should be interpreted with caution and requires further validation. Our finding is consistent with most investigation and further focus on a specific hypertension population. Among hypertensive patients, SUA also exerts complex non-linear effects on cognitive function, demonstrating both protective and detrimental impacts depending on concentration levels.

Specifically, we employed the SHAP value model to enhance model interpretability. Unlike other machine learning methods often criticized for their “black-box” nature, logistic regression is inherently interpretable. The integration with SHAP further equips it with dual explanatory power—both global and local (10). This not only demonstrates the average contribution of SUA to the occurrence of MCI but also elucidates how individualized input variables related to the outcomes for specific patients. This capability for individual case explanation is crucial for clinical decision support, allowing physicians to understand the rationale behind the model’s predictions for specific cases, thereby increasing their trust in the model’s outcomes. While SHAP can answer whether SUA is important, it is less suited for precisely delineating how it is important. RCS perfectly addresses this gap. By fitting smooth curves, it visually reveals the non-linear relationship morphology between SUA and MCI risk and can accurately identify critical inflection points (26). The specific inflection points values provided by RCS offer a concrete, data-driven target for potential clinical interventions. According to our RCS results, a ‘U-shape’ relationship between SUA and MCI implied that it may be beneficial to maintain a hypertensive and hyperuricemia patient’s SUA levels in a high-normal range for dementia prevention.

Additionally, this study found that there is an interaction between uric acid and a history of diabetes and regular exercise. A survey report found that older adults with diabetes and HbA1c ≥ 7.0% had a 38% increased risk of cognitive impairment (27). In individuals with diabetes, who often have underlying endothelial dysfunction and elevated oxidative stress (28), the antioxidant properties of UA might be particularly crucial. Therefore, the protective effect of moderate UA could be more pronounced in this high-risk subgroup. Regular exercise is known to improve cerebrovascular health, insulin sensitivity, and reduce oxidative stress. Implementing a scientifically informed fitness diet in conjunction with appropriate exercise may decrease SUA (29). In sedentary individuals, who lack these exercise-induced benefits, UA’s antioxidant role might become a more critical modifiable protective factor. Also, age, education level, albumin and thyroxin were found to be more important factors related to the occurrence of MCI than uric acid. Age is the most significant and irreversible risk factor for MCI. Aging is the most well-established and strongest risk factor for cognitive impairment and Alzheimer’s disease. All major global studies consistently show that the prevalence and incidence of cognitive impairment increase exponentially with age (30). After the age of 65, the risk of developing the disease nearly doubles every 5 years (31). Advanced age itself leads to known associations with the biological hallmarks, such as genomic instability, telomere attrition, epigenetic alterations, abnormal proteinosis, mitochondrial dysfunction and cellular senescence, leading to brain atrophy and function decline (32). Combined with factors such as hypertension, middle-aged and elderly populations should be key targets for cognitive impairment screening and prevention. Although age itself cannot be changed, understanding this risk can encourage individuals and healthcare systems to pay earlier attention to other modifiable risk factors and actively intervene to delay or mitigate the risks associated with aging.

Furthermore, education level is an important protective factor against cognitive impairment. This may be because higher education levels are believed to build cognitive reserve, thereby enhancing the brain’s resistance to pathological damage. Numerous studies have shown that low education level is a clear risk factor for cognitive impairment (33). Individuals with higher education may exhibit milder clinical symptoms or a later onset of symptoms even when some degree of Alzheimer’s pathology is present in the brain.

This study also found that albumin is a potentially modifiable risk indicator for cognitive impairment. Low albumin levels are associated with an increased risk of cognitive impairment (34). Albumin level is a key indicator of an individual’s overall nutritional status. Moreover, we found that weight, BMI and lipid metabolism are lower in MCI group, indicating nutritional status may relate to cognitive impairment. Malnutrition leads to insufficient albumin synthesis, and the brain requires continuous adequate nutrition and energy supply to maintain normal function (35). Malnutrition itself can directly impair cognitive function. Additionally, albumin has antioxidant and anti-inflammatory effects (36), which may counteract the significant oxidative stress and neuroinflammation accompanying neurodegenerative diseases. Low albumin levels imply a reduced ability of the body to resist these destructive processes, making neurons more vulnerable to damage. Albumin is able to bind to the precursor agent of the AD, amyloid-beta (Aβ) in the blood (34, 37), preventing its deposition in the brain and potentially slowing the pathological progression of Alzheimer’s disease. The link between albumin and MCI found in this study highlights the importance of nutritional screening and intervention for middle-aged and elderly hypertensive populations.

Several limitations of this study should be acknowledged. First, the relatively small sample size combined with the high incidence of MCI may introduce potential biases and affect the stability of the estimates. Future studies with larger cohorts are needed for validation. Second, the retrospective design and imbalanced distribution of samples may compromise the robustness of the findings. Additionally, the reliability of the data could have been influenced using self-reported health information. And the lack of detailed data on specific medications that may influence serum uric acid levels and cognitive function is a limitation. Future studies incorporating comprehensive medication data are needed to clarify these potential effects. Furthermore, the single-center origin of the data may limit the external validity and general applicability of our model. As Wenzhou is a coastal city, populations involved in this study may intake more high purine foods, which may influence the inflection point. Future prospective, multi-center studies with larger cohorts, balanced samples and more medication details are warranted to validate and extend our findings. Despite these limitations, this study provides an interpretable analytical approach to assess the role of uric acid in cognitive protection and help neurologists make more evidence-based clinical decisions.

## 5 Conclusion

In summary, by combining traditional logistic regression with both SHAP and RCS, this study demonstrates that serum uric acid (SUA) possesses a significant predictive value for mild cognitive impairment (MCI) among middle-aged and elderly hypertensive populations. The integration of SUA with other readily accessible clinical parameters—such as age, education level, and albumin—further enhances the accuracy of MCI risk assessment. Moreover, the results imply that maintaining SUA levels within an optimal range could serve as a feasible strategy to mitigate the risk of MCI in hypertensive patients. This insight opens avenues for future interventions aimed at modulating SUA concentrations, potentially through dietary or pharmacological means, to support cognitive health in vulnerable populations. Further prospective studies and randomized controlled trials are warranted to validate these observations and to elucidate the underlying mechanisms linking SUA, vascular health, and cognitive function.

## Data Availability

The raw data supporting the conclusions of this article will be made available by the authors, without undue reservation.
